# In Search of a Feedback Signal for Closed-Loop Deep Brain Stimulation: Stimulation of the Subthalamic Nucleus Reveals Altered Glutamate Dynamics in the Globus Pallidus in Anesthetized, 6-Hydroxydopamine-Treated Rats

**DOI:** 10.3390/bios13040480

**Published:** 2023-04-16

**Authors:** Mykyta M. Chernov, Christina B. Swan, James C. Leiter

**Affiliations:** 1 Department of Molecular and Systems Biology, Geisel School of Medicine at Dartmouth Medical School, Hanover, NH 03755, USA; 2 The White River Junction VA Medical Center, 215 N Main St, White River Junction, VT 05009, USA

**Keywords:** deep brain stimulation, subthalamic nucleus, globus pallidus, pseudorandom binary sequence, glutamate biosensor, Parkinson’s disease, system identification

## Abstract

Deep Brain Stimulation (DBS) of the subthalamic nucleus (STN) is a surgical procedure for alleviating motor symptoms of Parkinson’s Disease (PD). The pattern of DBS (e.g., the electrode pairs used and the intensity of stimulation) is usually optimized by trial and error based on a subjective evaluation of motor function. We tested the hypotheses that DBS releases glutamate in selected basal ganglia nuclei and that the creation of 6-hydroxydopamine (6-OHDA)-induced nigrostriatal lesions alters glutamate release during DBS in those basal ganglia nuclei. We studied the relationship between a pseudo-random binary sequence of DBS and glutamate levels in the STN itself or in the globus pallidus (GP) in anesthetized, control, and 6-OHDA-treated rats. We characterized the stimulus–response relationships between DBS and glutamate levels using a transfer function estimated using System Identification. Stimulation of the STN elevated glutamate levels in the GP and in the STN. Although the 6-OHDA treatment did not affect glutamate dynamics in the STN during DBS in the STN, the transfer function between DBS in the STN and glutamate levels in the GP was significantly altered by the presence or absence of 6-OHDA-induced lesions. Thus, glutamate responses in the GP in the 6-OHDA-treated animals (but not in the STN) depended on dopaminergic inputs. For this reason, measuring glutamate levels in the GP may provide a useful feedback target in a closed-loop DBS device in patients with PD since the dynamics of glutamate release in the GP during DBS seem to reflect the loss of dopaminergic neurons in the SNc.

## 1. Introduction

Patients in later stages of Parkinson’s Disease (PD) in whom tremor, postural instability, bradykinesia, or rigidity are poorly controlled by medical therapy become candidates for deep brain stimulation (DBS), a procedure in which some of the motor symptoms of PD, especially rigidity and tremor, are alleviated by chronic high frequency stimulation of specific basal ganglia nuclei, often the globus pallidus interna (GPi) or subthalamic nucleus (STN) [1,2]. The rationale for DBS is derived from a functional model of the basal ganglia that places the substantia nigra pars compacta (SNc) within a cortex-basal ganglia-thalamus-cortex motor loop and assigns to dopamine a critical role in modulating the signals between the striatum (the input structure of the basal ganglia) and the downstream nuclei of the basal ganglia, such as the globus pallidus (GP), the subthalamic nucleus (STN), and the substantia nigra pars reticulata (SNr) [3]. Based on this model, the lack of striatal modulation by dopaminergic neurons in the SNc in patients with PD results in inappropriate disinhibition of the downstream nuclei, increased GABAergic drive to the thalamus, and suppression of the flow of information from the thalamus to the cortex [3,4].

Therefore, surgical therapies for PD that emerged focused on suppression of the abnormal output of the downstream nuclei in the basal ganglia. For example, surgical ablation of the STN and the GP in PD patients appeared to restore an effective flow of information between the thalamus and cortex so that some patients experienced dramatic improvements in motor function after ablation of the STN or GP [5]. The use of microelectrodes to localize surgical targets for ablation led to the fortuitous discovery that high frequency stimulation of the thalamus alleviated tremor just as effectively as the subsequent surgery [6]. Deep brain stimulation has now largely replaced surgical resection as the surgical therapy of choice for PD, since the procedure is reversible, and the area targeted can be adjusted by repositioning the electrodes during surgery or adjusting the stimulation intensity or frequency, or increasingly, by redirecting or steering the direction of the stimulus after surgical implantation to improve symptom control or reduce side effects [7]. 

Despite the capacity to optimize DBS by adjusting the simulation settings, there is no consensus on the best strategy to achieve the optimal balance between symptom control and side effects. The “tried and true” method is for the treating physician to optimize control of symptoms during serial outpatient visits over months based on the balance of benefit versus unwanted side effects of DBS. Once the optimal stimulation waveform has been chosen, it is programmed into the stimulator, and the device operates in an open-loop mode with no feedback from the brain—the stimulation is dissociated from any of its immediate effects within the central nervous system. Numerous surrogate indices to try to improve the optimization process have been suggested, usually based on electrophysiological signatures of the circuit pathology of PD in the STN or GPi (e.g., the burstiness of neuronal activity in the STN or the spectral characteristics of local field potentials (LFPs) in the STN or other emergent electrophysiological patterns in the LFPs), but no single surrogate measure is clearly better than the tried and true method across the spectrum of parkinsonian symptoms [8,9,10].

DBS modifies neurotransmitter levels throughout the basal ganglia [11,12,13,14,15,16,17], and we and others have been testing the hypothesis that neurotransmitter release elicited by DBS might be used to create a closed-loop DBS device [11,18]. Therefore, the aim of the present study was to establish criteria that may be used to select candidate neuroanatomical sites as sources of a neurotransmitter-based feedback signal to control DBS in the STN. The STN is the most frequent target for DBS to ameliorate symptoms of DBS, and the STN contains glutamatergic neurons, which project to the GP in rats [19]. Hence, we used a pseudorandom binary sequence (PRBS) of pulses of DBS to characterize the dynamics of local glutamate release in the STN or in the GP during DBS of the STN in anesthetized rats. We performed simultaneous DBS and glutamate recordings in vivo using an amperometric biosensor. We convolved the PRBS stimulus train (the input) with the time varying concentration of glutamate in the STN or GP (the output) to create a transfer function, which is a mathematical description of the dynamical relationship between the pattern of DBS at a site (the stimulus) and the glutamate concentration measured in a given target nucleus (the response). Knowing the relationship between DBS and glutamate levels allows one to design a closed-loop DBS system in which the glutamate concentration can be used as a feedback signal to control DBS in order to maintain any given level of a neurotransmitter in a particular nucleus [11]. 

The quantitative relationship between DBS and glutamate levels in the basal ganglia has not been defined in either normal animals or animal models of PD. Therefore, we tested the hypotheses that DBS in the STN increases release of glutamate in the STN and GP and creation of 6-OHDA-induced nigrostriatal lesions alters the patterns of glutamate levels during DBS in the STN and GP in anesthetized rats. We measured glutamate levels in the STN and the GP during DBS in animals with and without 6-OHDA lesions to determine whether loss of dopaminergic neurons resulted in different transfer functions between DBS in the STN and glutamate concentrations in the STN and GP. 

## 2. Materials and Methods

The experiments were performed in rats according to NIH guidelines for animal use and were approved by the Institutional Animal Use and Care Committee at Dartmouth College. Male Sprague-Dawley rats weighing 80–100 gms (*n* = 19) were purchased from Charles River labs and housed at the institutional animal care facility in a temperature-controlled room (21 °C) with a 12/12 light cycle and with unlimited access to food and water. The rats were studied when they reached weights of 100–150 gms. 

### 2.1. Overall Study Design

In order to examine the effect of 6-OHDA lesions on glutamate release in the GP and STN during high frequency stimulation (HFS) of the STN, we first created hemi-parkinsonian animals by making unilateral 6-OHDA injections in the median forebrain bundle (MFB). We created the sham control animals by injecting vehicle alone unilaterally into the MFB in a different set of rats. Approximately three weeks after the 6-OHDA injections, we anesthetized each animal, placed a bipolar stimulating electrode in the STN and a glutamate biosensor in either the GP or the STN (within the STN, the biosensor and the bipolar electrode were placed simultaneously and adjacent to each other). During the actual test condition, we used a PRBS of HFS in the STN while recording the glutamate concentration in the GP or STN [11]. From these HFS-glutamate stimulus–response recordings, we calculated individual transfer functions for each animal and an average transfer function for each recording site (GP or STN ispi- or contralateral to the site of HFS in the STN).

Creation of Hemi-Parkinsonian Rats Using 6-OHDA

We injected 6-OHDA unilaterally into the region of the medial forebrain bundle to lesion dopaminergic neurons in the substantia nigra pars compacta [20]. Each animal received desipramine 25 mg/kg i.p. 30 min before the procedure. Each rat was anesthetized using an intraperitoneal injection of ketamine and xylazine (90 mg/kg and 10 mg/kg, respectively) and placed in a stereotaxic frame. The skull was exposed, and a 1 mm diameter hole was drilled on one side of the skull through which a 29-gauge needle connected to a microsyringe was guided. The tip of the injection needle was located stereotaxically 2.3 mm posterior and 1.5 mm lateral to bregma and 8.5 mm deep with respect to the dural surface. Each animal was injected with a 4 μL bolus of saline containing 4 mg ascorbic acid and 26 mg 6-OHDA (the treated group), or 4 mg ascorbic acid alone (sham-treated group). The injection rate was 1 µL/min, and the needle was left in place for 3 min after the injection and withdrawn slowly. The incisions on top of the head were sutured closed, and the animals were allowed to recover for 2–3 weeks. Over this time, the subset of the animals that received an injection of 6-OHDA developed unilateral lesions of dopaminergic neurons, which were confirmed by immunohistochemical staining for tyrosine hydroxylase at the conclusion of the study. 

### 2.2. DBS Stimulation in Anesthetized Animals

Two to three weeks after the injection of 6-OHDA or sham injection, each rat was anesthetized and placed in a stereotaxic frame. A concentric bipolar, platinum/iridium, stimulating electrode (O.D. = 200 µm; style CB-BFE75, FHC, Inc. Bowdoin, ME, USA) was inserted into the STN using the following stereotaxic coordinates: 3.5 mm posterior and 2.4 mm lateral with respect to bregma and 7.5 mm deep to the dural surface. A glutamate biosensor (O.D. = 180 µm at the tip; Pinnacle Technology, Inc., Lawrence, KS, USA) was placed initially on the same side of the brain as the stimulating electrode either in the STN or the GP. In the case of the STN, the stimulating electrode and the biosensor were affixed to each other, separated only by a small gap to prevent the two from coming into direct physical contact. In the case of the GP, the recording electrode was stereotaxically placed in a location 1 mm posterior and 3 mm lateral with respect to bregma and 7 mm below the dural surface, thereby placing the glutamate sensor in the mid-lateral GP. We also measured glutamate levels in the contralateral STN and GP in each animal by placing the glutamate biosensor at the mirror image stereotaxic site.

The STN was subjected to high frequency electrical stimulation using a pseudorandom binary sequence (PRBS) of stimulation pulses [11], and the glutamate concentration was recorded simultaneously in either the STN or GP at a rate of 1 sample per second. The PRBS consisted of a randomized sequence of fifteen 90 s periods during which the stimulator was either “on” or “off”. The PRBS does not simulate any clinical pattern of DBS, but was selected because the PRBS simulates white noise, which contains the optimal stimulation frequency characteristics for estimation of a transfer function using System Identification [21]. We titrated the stimulation intensity in each animal to the lowest level necessary to elicit a small rise in glutamate after a short square wave of stimulation at 150 Hz. Once a suitable intensity of stimulation was selected in each animal, the same intensity was used consistently throughout each PRBS of stimulation in that animal. When the stimulator was on during the PRBS, the STN was stimulated with a train of monophasic square-wave electrical pulses 60 μS in width at 150 Hz with a current intensity of 40–200 μA. During the “off” periods in the PRBS, the stimulator was in high impedance mode. A LabVIEW (National Instruments, Austin, TX, USA) virtual instrument was used to generate the stimulation signal and simultaneously plot the glutamate concentration. After the PRBS was completed, the electrodes were placed on the other side of the brain and the stimulation was repeated. The average current intensity was not different between the 6-OHDA-treated and the control animals, and the estimated transfer function was not affected, so far as we have been able to determine, by the number of elements, duration, or intensity of PRBS stimulation [11].

### 2.3. Intracranial Measurement of Glutamate Levels during DBS

Glutamate measurements were performed using an enzyme-based biosensor (Pinnacle Technologies, Inc., Lawrence, KS, USA). The sensor was coated with glutamate oxidase, which in the presence of oxygen, rapidly converts glutamate into alpha-ketoglutarate and hydrogen peroxide. The hydrogen peroxide was detected electrochemically using a platinum wire electrode at a holding potential of 0.4 V. The current flow related to oxidation of hydrogen peroxide is proportional to the glutamate concentration in the electrode cavity. The sensors were calibrated according to the manufacturer’s instructions by recording the current generated in a set of glutamate solutions of known concentration before and after in vivo implantation. The normal levels of glutamate under unstimulated conditions are just above the limits of detection of the amperometric electrodes. The electrodes are better suited to measuring changes in glutamate levels (above baseline values) during stimulated conditions than estimating the average tissue glutamate level in the basal state, and this is the way we used them in this study.

### 2.4. Verification of Electrode Placement

**Figure 1:**
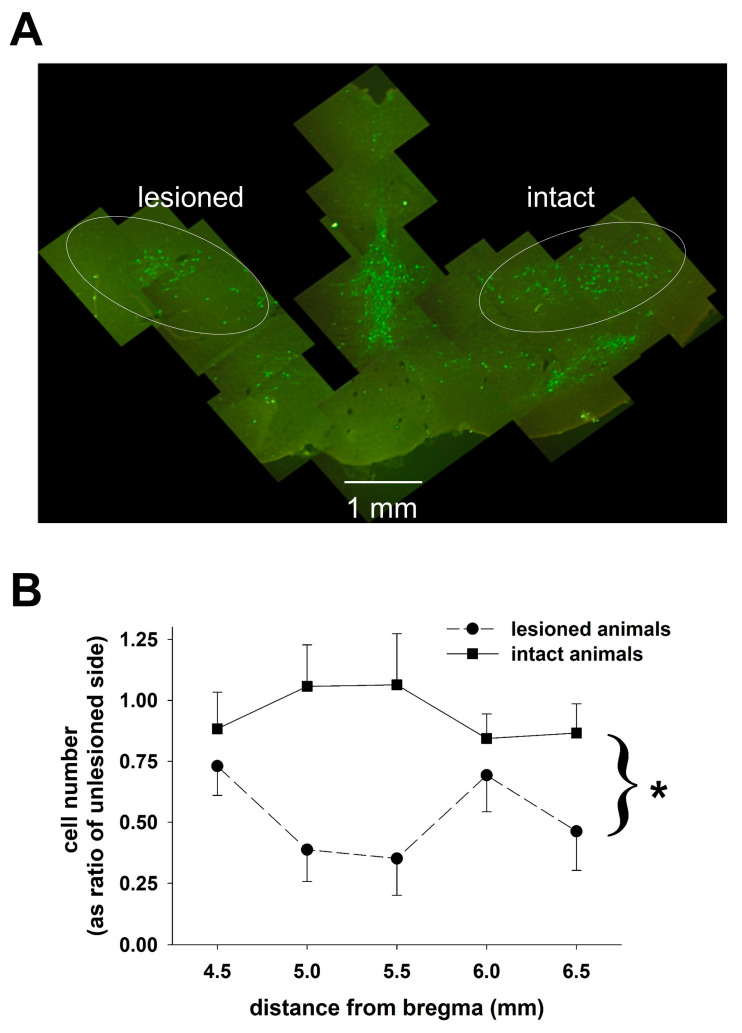
Effect of the 6-OHDA treatment on the number of tyrosine hydroxylase-positive cells in the substantia nigra pars compacta. We could not simultaneously capture an entire coronal section containing both left and right SNc from a single image and still retain the magnification that shows individual cells clearly. Therefore, we stitched multiple smaller, higher magnification images together—all taken from a single coronal brain slice—to create the representative composite image of tyrosine hydroxylase staining shown in panel (**A**) from a single animal that received a unilateral 6-OHDA injection into the MFB. The approximate location of the SNc has been indicated by the ovals on the image. Note the relative lack of tyrosine hydroxylase-positive cells on the left side of the image within the oval on the lesioned side. In the lower panel (**B**), the average ± SEM number of tyrosine hydroxylase-positive images along the rostro-caudal extent of the SNc has been compiled from all the 6-OHDA treated and untreated animals both ipsilateral and contralateral to the 6-OHDA injection in each animal. The number of tyrosine hydroxylase-positive cells has been expressed as the ratio of the number of tyrosine hydroxylase-positive cells on the injected side to the untreated side. A ratio of one indicates no difference in the numbers of tyrosine hydroxylase-positive cells comparing the sham- or 6-OHDA-injected side to the non-injected side. The ratio hovers near one in the sham-treated animals and is significantly less than one in the 6-OHDA-treated animals since the cell loss was asymmetrical in the 6-OHDA-treated animals. There was no apparent regional variation in tyrosine hydroxylase-positive cell loss as a function of distance from the bregma, and the 6-OHDA treatment substantially reduced the number of tyrosine hydroxylase-positive cells on the side ipsilateral to the 6-OHDA injections (a main effect of 6-OHDA treatment side, *p* < 0.001 indicated by the “*”).

**Figure 2:**
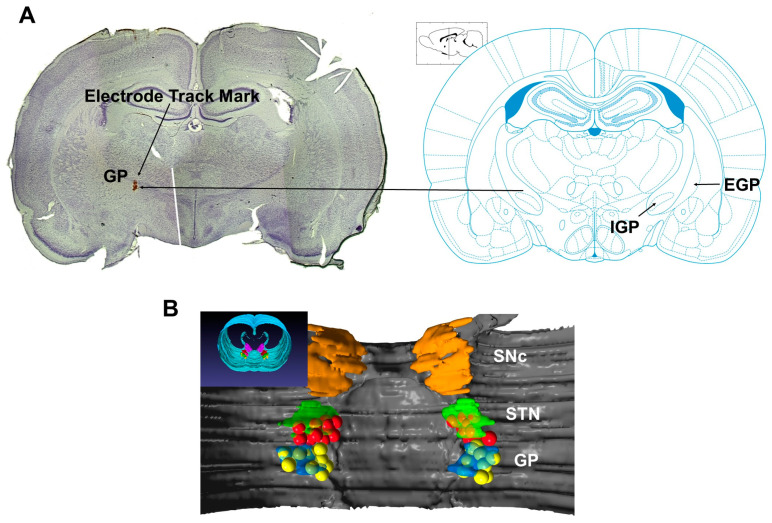
Neuroanatomical locations of stimulation electrodes and glutamate biosensors. A representative photomicrograph of a cresyl violet-stained section of the brain (**A**) shows the location of the tip of the biosensor in the GP on the left panel (arrow pointing to brown discoloration), and a coronal neuroanatomical schematic (right panel) showing the location of Internal GP (IGP) and the External GP (EGP) located −2.28 mm from the bregma (adapted from Paxinos and Watson, Plate 52, 5th Ed.). Note the discoloration associated with the track made by the biosensor just lateral to the IGP. In the lower panel (**B**), a composite three-dimensional image has been created to show the sites of stimulation in the STN and the sites of each glutamate biosensor in the STN (red spheres) or in the GP (yellow spheres). The inset shows the outline of the brain and nuclei derived by creating a z-stack of images (rostral at the front/bottom; caudal at the back/top of the images) from an anatomical atlas [22]. The SNc, the STN, and the GP are highlighted to show the anatomical relationships among the nuclei. This is a selected sample of electrode and biosensor sites (indicated by colored spheres) featuring only those stimulation and biosensor recording sites in which a significant relationship between stimulation in the STN and glutamate release in the target nucleus was achieved (a transfer function with an r^2^ value > 0.9). Stimulation or biosensor locations outside the regions shown in this figure are not shown since there was no significant glutamate release detected during putative stimulation of the STN in these studies (three animals were excluded because the biosensor was not in the GP; two animals were excluded because the biosensor was not in the STN; and three animals were excluded because the stimulating electrode was not in the STN). Thus, there was a tight relationship between effective stimulation of the STN and detection of glutamate release in the target nuclei, which is reflected in the dense clustering of those stimulation and biosensor locations associated with meaningful transfer functions within or very closely adjacent to the target nuclei.

Following the experiment, each animal was deeply anesthetized with ketamine/xylazine and perfused with saline, followed by perfusion with 4% paraformaldehyde in phosphate buffered saline (PBS). The brains were removed and placed in 4% paraformaldehyde for 48 h followed by cryoprotection in a solution of 30% sucrose. The brains were then frozen, embedded in O.C.T. medium, and cut into 50 µm sections. Alternate slices were stained with cresyl violet for verification of electrode placement or saved for immunohistochemistry (see Figure 1). Electrode locations were established from the track marks left in the tissue during implantation using a brain atlas [22]. Additionally, a three-dimensional reconstruction of the STN, the GP, and the substantia nigra pars compacta was made based on the atlas of Watson and Paxinos [22], and the identified locations of the tips of the electrodes were placed within the relevant neuroanatomical structures in this reconstruction (see Figure 2). 

### 2.5. Determination of 6-OHDA-Induced Dopaminergic Neuronal Loss

Fixed tissue, prepared as described above, was obtained from four animals treated with 6-OHDA and four sham-treated animals. Coronal sections, ~50 microns thick, were cut through the region of the SNc using a cryostat, and sections were collected approximately every 0.75 mm moving rostral to caudal through the SNc so that 5–8 sections were obtained for cell counting from each animal. To stain sections of the brain for tyrosine hydroxylase activity, tissue sections were incubated in PBS containing monoclonal anti-tyrosine primary antibody (MAB 318, Millipore, Billerica, MA, USA). The sections were initially washed with 3% hydrogen peroxide and 0.1% Triton-X in 0.1 M PBS, pre-incubated in 5% normal goat serum and washed with 0.1 M PBS. The tissue was incubated with the primary antibody (1:400 dilution) for 48 h at 4 °C, washed with PBS and incubated with the secondary antibody, Cy2-conjugated goat anti-mouse antibody (AP124J from Millipore at a 1:200 dilution), for 2 h. Each section was examined using a conventional fluorescence microscope with a motor driven stage. Commercially available software (Stereo Investigator, MBF Bioscience) was used to estimate the numbers of tyrosine hydroxylase-positive neurons on both sides of the brain in the region containing the SNc using the non-biased optical fractionator technique.

### 2.6. Data and Statistical Analysis

We used System Identification to derive a transfer function between the PRBS of high frequency stimulation and glutamate levels in the STN or GP [21]. In modeling the dynamic changes in glutamate levels as a function of DBS, we assumed that the relationship between HFS and glutamate levels was linear, causal, and time invariant, assumptions that seem warranted based on previous studies [11]. Such a system can be estimated using a linear difference equation, where the weighted sum of all inputs plus a weighted error or disturbance term is equal to the weighted sum of all outputs:

y(t) + a_1_y(t − 1) + … + a_n_y(t − n_a_) = b_1_u(t − 1) + … + b_n_u(t − n_b_) + c_1_e(t − 1) + … + c_n_e(t − n_c_)

The goal of the linear regression algorithm is to find the appropriate set of weighting factors a, b, and c to balance the two sides of the equation [21]. The model that we used was an Autoregressive Moving Average Exogenous (ARMAX) model, which is a more general form of the Autoregressive–Regressive Exogenous (ARX) model that we used in an earlier study [11]. The ARMAX model treats the error term as a moving average of white noise rather than assigning it a fixed weight as in the ARX model [11], and white noise seemed like a more appropriate representation of the biological variation represented by the error term. The model can be rewritten in a more compact form by using the delay notation in which the powers of the delay term q correspond to the number of samples before the current point in the sequence incorporated into the estimate of the current value (the incorporation of information from samples before or after the current point constitutes the autoregressive aspect of the model), so that q^−1^u(t) is equivalent to u(t − 1):

A(q)y(t) = B(q)u(t) + C(q)e(t)

Thus, the transfer function for the deterministic part of the process is G(q,θ) = B(q)/A(q), and the transfer function for the stochastic part of the process is

G(q,θ) = C(q)/A(q)

where θ is the matrix of the weighting factors a, b, and c. We used a pseudorandom binary sequence as our input, taking advantage of the fact that in a linear system, an appropriately designed PRBS is equivalent to an impulse input, an input that contains band-limited white noise, and therefore tests the system response across the full range of system dynamics and produces a response that is a complete description of the system dynamics [23]. The PRBS contains sufficient variation of both “on” and “off” periods of stimulation to capture both the rise times and decay times of glutamate levels within the transfer function. The System Identification Toolbox of Matlab (The Mathworks, Inc., Natick, MA, USA) was used to generate an ARMAX model relating the PRBS stimulation sequence to the measured glutamate concentration. The transfer function generated by the model was considered adequate if the least squares fit between the predicted and the measured responses to the PRBS had a correlation coefficient (r^2^) > 0.9. The ARMAX model provides standard error estimates for each parameter in the model, but these estimates are not easily graphed or interpreted since the number of parameters is relatively large, and the error variation in any single parameter contributes relatively little to the overall quality of the fit of the model to the experimental data. Therefore, we used the r^2^ value as the best, single estimate of the overall model fit.

## 3. Results

### 3.1. Confirmation of 6-OHDA Efficacy and Electrode Placement

Injections of 6-OHDA into the MFB resulted in lesions of dopaminergic neurons in the substantia nigra pars compacta. Figure 1, top panel, is a composite micrograph of dopaminergic neurons stained with tyrosine hydroxylase taken from a single coronal section through the SNc from a single animal that demonstrates a reduction in the number of tyrosine hydroxylase-positive cells ipsilateral to the injection of 6-OHDA in the MFB. The stereological dissector method was used to count stained cells on each side of the brain in order to construct the graph in the lower panel in Figure 1, which shows the average ± SEM number of tyrosine hydroxylase-positive images along the rostro-caudal extent of the SNc compiled from all of the 6-OHDA-treated and intact, sham-treated animals both ipsilateral and contralateral to the injection site in the MFB in each animal. We compared the ratios of tyrosine hydroxylase-positive cells on the lesioned side to the unlesioned side in each animal in the 6-OHDA-treated and in the sham-treated animals in a two-way ANOVA: treatment group (6-OHDA or sham, a between-subjects factor) by distance from the bregma (a repeated, within-subject factor). There was no significant interaction in the two-way ANOVA (F_(4,24)_ = 2.19; *p* > 0.10), and no significant effect of the distance from the bregma (F_(4,24)_ = 1.71; *p* > 0.18). The only statistically significant *p*-value was the main effect of the treatment group (F_(1,6)_ = 36.86; *p* < 0.001), which indicates that, across the length of the SNc, there was a homogenously reduced ratio of tyrosine hydroxylase-positive cells on the 6-OHDA-treated side compared to the untreated side in the 6-OHDA-treated animals, and similarly, a homogenously consistent and stable ratio of tyrosine hydroxylase-positive cells close to one in the sham-treated animals (no difference in tyrosine hydroxylase-positive cells on the sham-treated and untreated sides). The severity of the dopaminergic cell loss varied from animal to animal and never reached 100%. On average, ~45% (range 30–80%) of the dopaminergic neurons were lost within the SNc on the 6-OHDA-treated side of the brain compared to the unlesioned side of the brain in the same animal. Overall, these were mild lesions with only modest loss of dopaminergic neurons, which is to be expected in the relatively young animals studied.

The locations of the recording and stimulating electrodes were clearly visible on the cresyl violet-stained sections of the brain, such as the photomicrograph shown on Figure 2, upper panel. To create a composite figure of the electrode placement locations from all animals, we marked the locations of the electrode tips in a digitized copy of the rat brain atlas by Watson and Paxinos [22] and created a three-dimensional image by merging multiple pages of the atlas into a Z-stack and highlighting the relevant anatomical structures, such as the SNc (orange region), the STN (green region) and the GP (blue region). From the stained sections, it was clear in several animals that stereotaxic placement of either the stimulating or the recording electrode (or both) was unsuccessful. Recording and stimulating sites that were “on target” in the GP were marked with small yellow spheres, and the on-target recording or stimulating locations in the STN were marked with small red spheres. In the animals with off-target electrode placements, there was no detectable release of glutamate during stimulation (the maximal measured values were less than 10 µM during DBS) and attempts to fit the responses of these animals to the ARMAX model obtained in correctly implanted, on-target rats were unsuccessful. The correlation coefficients of the model fits in the animals with off-target electrode placements were all less than 0.7. Therefore, these animals were excluded from further analysis, and the electrode and biosensor locations are not shown in Figure 2; three animals were excluded because the biosensor was not in the GP; two animals were excluded because the biosensor was not in the STN; and three animals were excluded because the stimulating electrode was not in the STN). In no case did an animal with both the stimulating electrode and the biosensor in or near the anatomical target have a transfer function with an r^2^ < 0.7; the correlation coefficient of the ARMAX model was greater than 0.9 in all the animals in which the stimulating electrode and the recording biosensor were located in the anatomical targets. Thus, there was a tight relationship between effective stimulation of the STN and detection of glutamate release in the target nuclei, which is reflected in the tight clustering of those stimulation and biosensor locations associated with meaningful transfer functions in the target nuclei shown in Figure 2, lower panel; the yellow spheres and red spheres were in or closely adjacent to the GP or STN (the target nuclei), respectively.

### 3.2. An Example of Glutamate Dynamics during STN Stimulation and the Analysis Method

**Figure 3:**
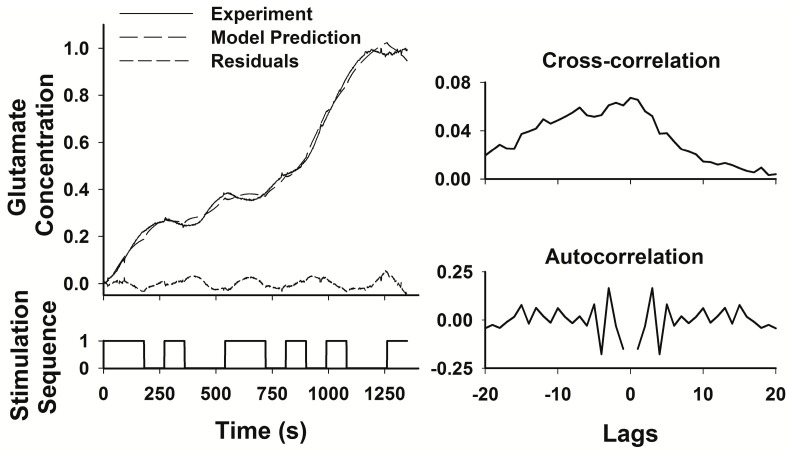
An example from a single animal of the dynamic changes in the glutamate concentration in the STN during DBS in the STN. The upper left panel shows the glutamate concentration as a function of time and the PRBS of DBS (shown below the glutamate concentration graph). The upper right panel shows the cross correlation between the model residual and the PRBS (there is no significant cross correlation), and the lower right panel shows the autocorrelation within the residuals, where lags are expressed as a function of the number of samples shifted (each sample represents 1 s). There was no significant autocorrelation. The fitted model effectively replicated the dynamics of glutamate levels within the STN, and the lack of any residual correlations in the model indicates that the variation in the experimental results not accounted for in the model probably represents stochastic noise.

Stimulation of the STN using the PRBS elicited robust and reproducible release of glutamate both in the STN itself and in the GP. An example of glutamate levels in the STN during DBS in the STN in a 6-OHDA-treated animal ipsilateral to the 6-OHDA injections is shown on Figure 3. The transfer function was obtained by fitting the data to an ARMAX model with 8 poles, 5 zeros, 5 error terms and a dead time of 1. The analysis of the goodness-of-fit of the model requires an evaluation of the residuals and the autocorrelation and cross correlation of the model residuals (Figure 3). The experimental results (black line), the fitted model prediction (dashed line), and the model residuals (dotted line) have been plotted as a function of time and the pseudorandom sequence of stimulation (bottom left panel). The fit between the measured glutamate concentration and the ARMAX prediction of the glutamate levels expressed as a function of the PRBS of stimulation was excellent (upper left panel). The cross correlation of residuals and the PRBS and the autocorrelation of residuals have been plotted as well. The plot of the model residuals (dashed line) was relatively flat, and the autocorrelation of the model residuals and cross correlation of the model residuals and the PRBS of stimulation did not reveal any significant correlations, which indicates that there is no residual, temporally varying information relating the stimulus (the PRBS of DBS) to the response (glutamate levels) that the model has missed. The remaining, unfitted variation quantified by the residuals likely represents stochastic noise. These results, therefore, confirm that the ARMAX model successfully captured the dynamics of the system [11]. 

Due to the complex pattern of the input, the nature of the transfer function relating high frequency stimulation of the STN to glutamate dynamics in the STN or GP cannot be gleaned from visual inspection of the data alone, and one must compare the transfer functions to evaluate glutamate responses to DBS in different nuclei and different experimental conditions.

### 3.3. Glutamate Dynamics in the GP during STN Stimulation

**Figure 4:**
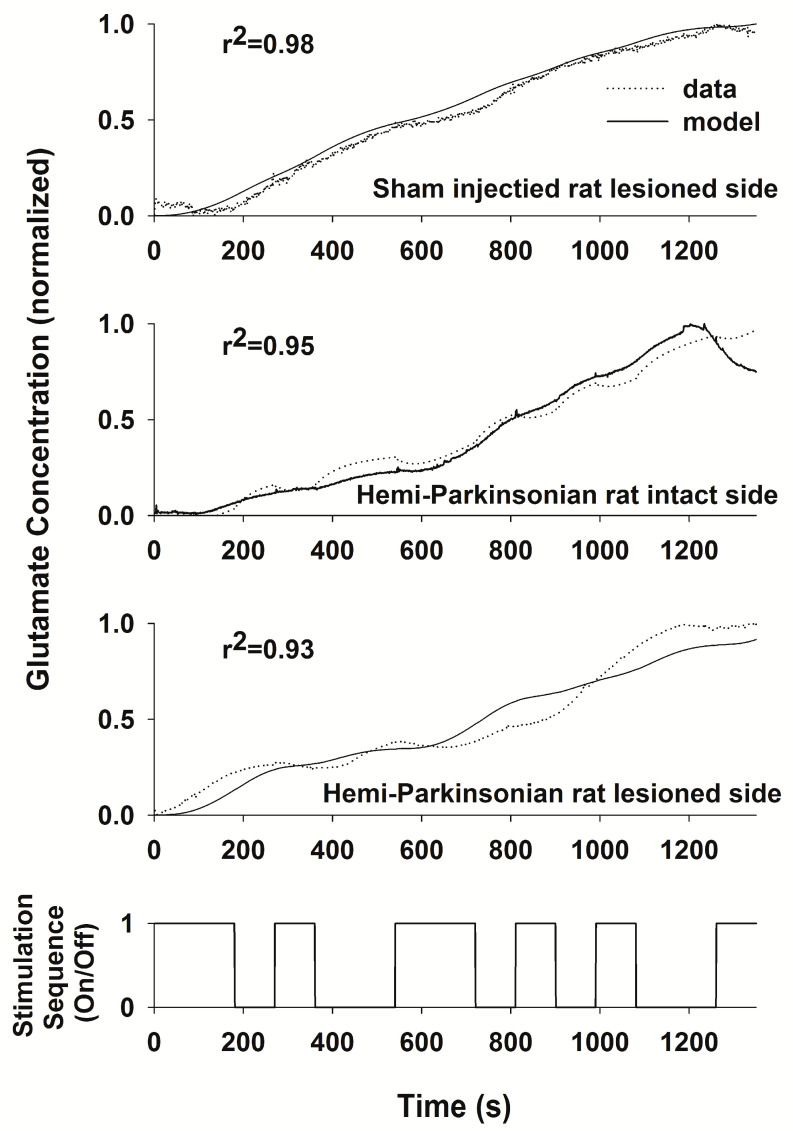
The average experimental results and model fits showing the dynamic variation of glutamate levels in the GP as a function of the PRBS of DBS in the STN and time in three groups of animals (the top three panels show the average glutamate response in each of the three treatment conditions, and the bottom panel shows the sequence of pseudorandom stimulation, where ‘1’ represent stimulation on, and ‘0’ represents stimulation off). For each group of animals, the fitted model had a high correlation, and though not apparent on visual inspection, the parameters of the three models were different. Moreover, the analysis of residuals and the cross- and autocorrelations indicated that there was no residual information that the ARMAX models missed in any treatment group or at any neuroanatomical site.

Average dynamic changes in glutamate levels in the GP during DBS in the STN are shown in Figure 4 for three treatment conditions. There is a lag between the onset of stimulation and changes in the glutamate concentration. Moreover, the concentration of glutamate reached a plateau by the end of the PRBS of stimulation. Although individual neurons may respond quickly to stimulation, the level of glutamate in the interstitial space surrounding the biosensor reflects a variety of processes: the space around the biosensor acts as a spatial buffer and slows the rise and fall of glutamate levels, the rates of glutamate release and uptake of glutamate within the GP, the rate of diffusion of glutamate from the site of release to the biosensor, and the distance of the biosensor from the site(s) of glutamate release all contributed to the temporal relationship between the DBS stimulation and the measured glutamate levels. The plateau in glutamate levels late in the stimulation sequence indicates that equilibrium was established among neurotransmitter release, glutamate reuptake, and diffusion from the site of release, which prevented any further increase in the glutamate concentration. 

To answer the question whether glutamate dynamics in the GP during STN stimulation were different between the hemi-parkinsonian animals and the sham controls, responses from multiple animals (*n* = 5 in each group) were averaged, and the average data were fitted using the ARMAX model as shown in Figure 4. We plotted the transfer functions between HFS in the STN and glutamate levels in the GP in three data sets: the responses of the sham control animals (results from the sham-injected and sham-non-injected side were combined since they were not different), the responses in the hemi-parkinsonian animals recorded on the side of the 6-OHDA-induced lesion and last, the response on the untreated side in the hemi-parkinsonian animals. Glutamate levels in the GP during DBS in the hemi-parkinsonian animals changed more sluggishly than did glutamate levels in the GP in the saline-injected control animals. Surprisingly, the rates of change in glutamate levels on the intact side of hemi-parkinsonian animals were also sluggish, and the glutamate response resembled the response on the lesioned side of the brain. 

**Figure 5:**
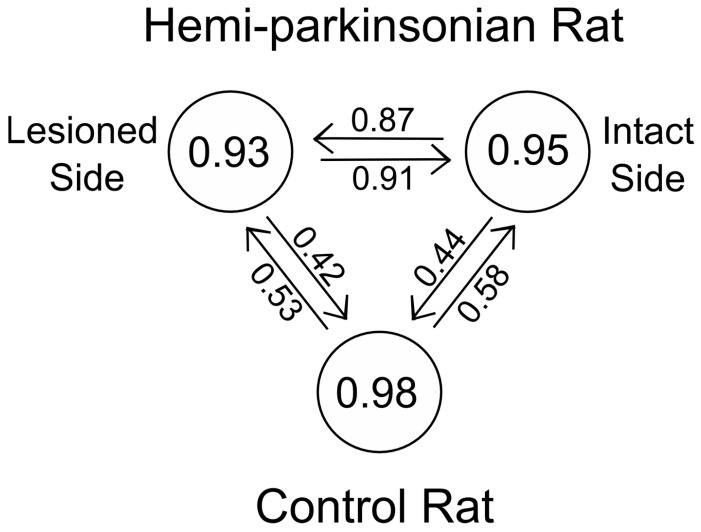
Correlation coefficients among the model fits derived from each experimental group in which glutamate levels were measured in the GP. The numbers within each sphere indicate the average correlation between glutamate levels and the fitted model at each neuroanatomical site, and the numbers associated with each arrow indicate the correlation derived from fitting a model derived from one set of studies to data derived from another set of studies. The models derived from the 6-OHDA-treated animals on the lesioned and intact side of the brain fitted the data from the other side of the brain surprisingly well (r^2^ = 0.87 or 0.91). In contrast, neither model derived from the 6-OHDA-treated animals fit the experimental data from the intact animals and vice versa.

The divergent patterns of glutamate dynamics during DBS of the STN in the different nuclei are described quantitatively by a set of ARMAX models (as shown in Figure 4), but there is no easy statistical test to detect significant differences among the models—each model has a large number of parameters, and a parameter by parameter comparison has low power to detect global differences among models given both the large number of parameters (each parameter represents only a small part of the global differences among treatment groups and recording sites) and the relatively small number of animals in each group. There were three experimental datasets created: the 6-OHDA-treated animals fitted on the lesioned and unlesioned side and the sham-treated animals, for which the treated and untreated sides were pooled as they were not different. We compared the global descriptive power of each model by a shuffling process in which the models for each of the three unique combinations of treatment group were applied to the two other unique combinations of treatment group and recording site on the assumption that if the models were significantly different, there should be a dramatic decline in the quality of the fit of the model to the data, as reflected in the global model output that we used to determine the goodness-of-fit—the r^2^ value. We fit the responses of each experimental group to its own ARMAX model using a transfer function that had the same number of terms and model structure whether applied to the lesioned or unlesioned animals; only the parameters of the models differed among the different treatment groups. The models provided a good correspondence to the observed experimental data from which they were derived (as they should: r^2^ > 0.9 for the models, as shown inside the circles in Figure 5). We created a pooled estimate of the 95% confidence interval of the correlation coefficients for the properly matched model output and experimental data, which was between ~ 0.71 and 0.96. Therefore, any r^2^ term from a model fit to the experimental data that was less than ~ 0.71 was a significantly worse fit than the fit derived from the correct matching of model and experimental data. The two transfer functions describing glutamate dynamics in the GP during PRBS stimulation in the STN on the lesioned and unlesioned sides in the hemi-parkinsonian rats were interchangeable—not detectably different from each other (note the similar correlation coefficients equal to 0.87 and 0.91, indicated by the arrows between each sphere). However, when we tried to fit the data from the untreated control animals to either model of the 6-OHDA-treated animals, the model fits were significantly degraded (r^2^ values < 0.6, well below the lower bound of the 95% CI for the “correctly” matched data and model results). Thus, a 6-OHDA-induced lesion on one side of the brain seemed to alter the dynamic changes in glutamate release and uptake during HFS of the STN on both the lesioned and unlesioned sides of the brain compared to the sham-treated control animals. 

### 3.4. Glutamate Dynamics in the STN during STN Stimulation

**Figure 6:**
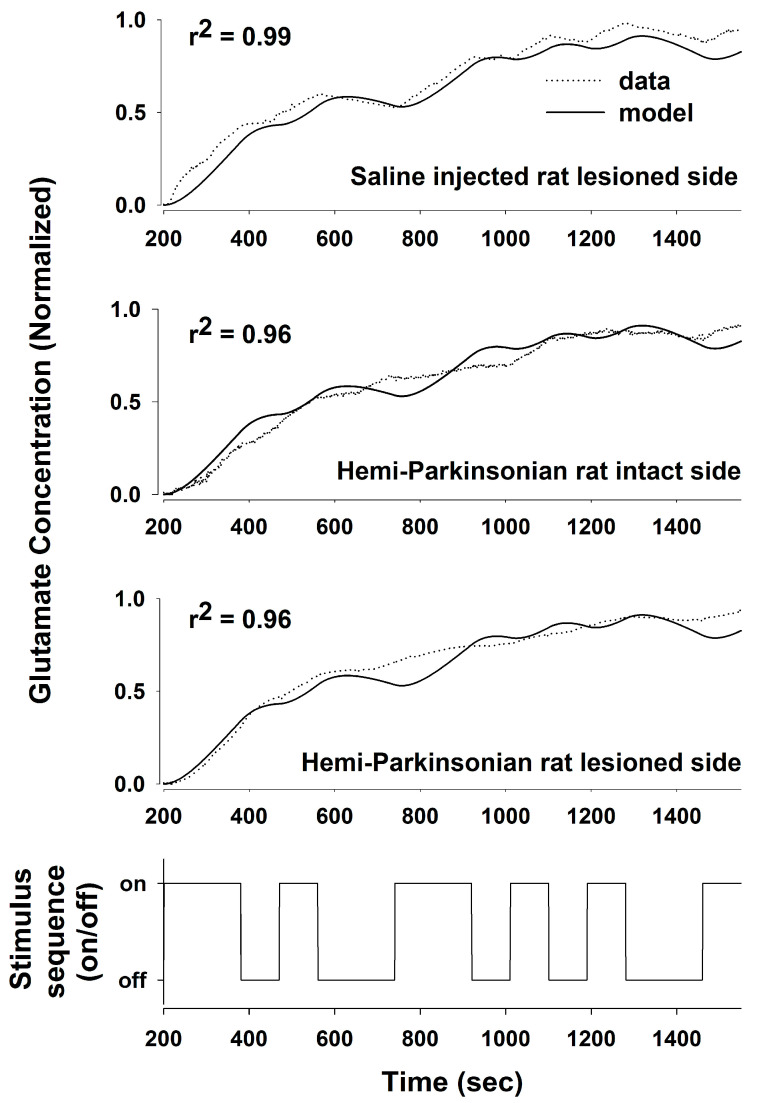
The average experimental results and model fits showing the dynamic variation of glutamate levels in the STN as a function of the PRBS of DBS in the STN and time in three groups of animals. For each group of animals, the fitted model had a high correlation, and unlike glutamate levels in the GP, the parameters of the three models were virtually identical—the presence or absence of 6-OHDA treatment did not change the parameters of the model significantly.

Stimulation of the subthalamic nucleus using the PRBS resulted in local release of glutamate within the STN, just as we demonstrated previously [11]. The average glutamate responses in each experimental condition are shown in Figure 6; the results of the sham-injected (*n* = 6) and 6-OHDA-injected animals (*n* = 5) were combined since they did not differ. Compared to glutamate levels in the GP, glutamate levels changed more quickly in the STN (note the absence of a lag in the rise of glutamate immediately after the PRBS of DBS started) and demonstrated local glutamate minima and maxima that tracked the switching of the PRBS of stimulation “on” and “off”. The destruction of dopaminergic neurons following injection of 6-OHDA did not alter glutamate release in the STN as shown in Figure 6. Not only do the responses from the saline-injected control animals and their hemi-parkinsonian counterparts look the same, but the dynamic changes in the levels of glutamate in the STN were predicted remarkably well either by the transfer function from the saline control or the hemi-parkinsonian animals. Thus, despite some variation in the small features of the measured glutamate levels among the treatment groups, the correlation coefficient was greater than 0.9 regardless of which transfer function (lesioned or sham control) was used to fit the data from each group of animals. In contrast to glutamate levels in the GP during DBS in the STN, DBS in the STN resulted in glutamate levels within the STN that were not dependent on dopaminergic neuronal input. 

## 4. Discussion

We used a PRBS of stimulation and System Identification to obtain a transfer function relating DBS stimulation in the STN to local changes in the glutamate concentration in the STN and in the GP in anesthetized rats. Using a PRBS and System Identification is a broadly applicable analytical method that is not constrained by the length or content of the particular PRBS used (they all simulate the frequency content of white noise) or responses being measured [24,25]. From the transfer functions that we obtained, we were able to define the stimulus–response characteristics of glutamate levels following HFS in the STN to address the question whether glutamate release during DBS is site-specific and whether it is altered in the GP or the STN following 6-OHDA-induced dopaminergic cell loss in the SNc. We obtained transfer functions that effectively described the stimulus–response relationships between DBS in the STN and glutamate levels in both the STN and GP and in both the sham control and the hemi-parkinsonian rats created by unilateral injection of 6-OHDA adjacent to the MFB. The dynamics of glutamate release during DBS differed in the GP and the STN. The concentration of glutamate in the STN rose quickly in response to HFS within the STN and tracked the “on” and “off” states of the stimulation sequence well, although the declines in the glutamate concentration during each “off” phase were not fast enough to prevent the extracellular glutamate concentration from reaching a plateau toward the end of the PRBS of stimulation. The response in the GP, on the other hand, was more sluggish, and the glutamate concentration did not start rising appreciably until about 150–200 s after the start of the stimulation. The best fit transfer functions between DBS and glutamate concentrations in the GP and the STN were also different. The relationship between DBS and glutamate levels in the STN can be described quite accurately with a second-order transfer function [11]. In order to describe accurately the dynamics of glutamate release and uptake in the GP, we had to expand our model to an 8th order system; longer time lags were needed to make the model fit the dynamical behavior of the larger network of neurons that intervenes between the site of stimulation in the STN and the site of glutamate release and uptake in the GP. Describing glutamate levels in the STN with an 8th order system resulted in little additional increase in the goodness-of-fit of the regression to the data over the simpler, 2nd order system that we described previously [11]. The loss of dopaminergic neurons was relatively subtle in this study, likely because the animals were young at the time of the 6-OHDA injections. Nevertheless, the transfer functions derived from a PRBS of HFS in the STN effectively differentiated among the pattens of glutamate levels in the STN and GP, even when the loss of dopaminergic neurons was probably below the threshold of clinically significant dopaminergic neuronal loss (80–85% of dopaminergic cell loss is usually present in the substantia nigra when symptoms of PD appear in humans) [26,27]. Thus, the transfer functions developed using System Identification provided a sensitive analytical tool to describe the relationships between HFS in the STN and glutamate levels in two target nuclei. 

These results point to fundamental differences in the mechanisms of DBS-induced neurotransmitter release in the two sites studied. The fast dynamics of glutamate release in the STN are probably due to antidromic stimulation of afferent glutamatergic fibers originating in layer V of the motor cortex or activation of local interneurons within the STN, although the latter possibility is less likely, given the tendency of DBS, with its extremely short pulse width (60 μs), to target axon tracts rather than cell bodies [28,29]. In contrast, the relationship between HFS in the STN and glutamate levels in the GP was smeared in time, and the more sluggish changes in glutamate levels in the GP may be due to a more indirect, polysynaptic neural route followed by the stimulating impulse originating in the STN before the effect of stimulation arrives at the GP, although the precise path of influence between the STN and GP during DBS is not clear, since there may be retrograde and antegrade effects of DBS.

Destruction of dopaminergic neurons in the substantia nigra did not alter glutamate release within the STN during DBS of the STN. In light of the importance placed on the role of the hyperdirect pathway between the STN and the cortex in Parkinson’s Disease, this finding may be somewhat surprising. Even though a dopaminergic lesion in the SNc may change the intrinsic firing rates and pattern of firing of the glutamatergic neurons in the STN, this is due to alterations in the brain circuit somewhere upstream or downstream of the striato-subthalamic glutamatergic synapse. It seems that DBS in the STN dominates the local circuits controlling glutamate levels in the STN, and as a result, glutamate levels in both the intact and lesioned animals were similar and unaffected by dopaminergic cell loss from the more distant elements of the basal ganglia circuit. On the other hand, the dynamics of glutamate levels in the GP were different comparing the saline-injected control animals and the 6-OHDA-treated animals, indicating that dopaminergic neurons in the SNc are more directly involved in the circuits controlling glutamate levels in the GP. Thus, the strategy of stimulating the STN while measuring glutamate in the GP is better suited to assessing changes induced by the 6-OHDA lesion since glutamate levels in the GP seem to depend on those parts of the basal ganglia circuit that are being affected by dopaminergic inputs from the SNc, whereas glutamate levels within the STN seems to be dependent more on local conditions within the STN than on the state of the circuits distributed more widely in the basal ganglia.

Unilateral dopaminergic lesions had a bilateral and equivalent effect on glutamate levels in the GP. We are not the first to note that unilateral 6-OHDA lesions and unilateral stimulation of the STN may produce bilateral effects. Unilateral 6-OHDA lesions increase glutamate bilaterally in the caudate-putamen of rats, and the increase in the glutamate concentration is surprisingly similar ipsi- and contralateral to the 6-OHDA lesion [30]. Bilateral changes in glutamate transporter 1 expression and activity [31] and in vesicular glutamate transporters [32] have also been described in the 6-OHDA-treated hemi-parkinsonian animals. HFS of the STN also produced bilateral reductions in neuronal firing within the STN [33]. Moreover, HFS of the STN caused approximately equal increases in dopamine concentrations in the striatum ipsi- and contralateral to the site of stimulation [34,35]. There are a small number of crossed nigrostriatal fibers in rats [36], and there may be thalamic or cortical decussating fibers that make these bilateral enzymatic, neurotransmitter, and electrical changes possible following unilateral 6-OHDA lesions and during unilateral HFS of the STN. That a single transfer function described glutamate concentrations in the GP and STN so effectively during unilateral HFS of the STN in the hemi-parkinsonian animals should not have come as a surprise to us in light of the foregoing studies; nonetheless, the goodness-of-fit of a single transfer function to the stimulus–response relationships on both the lesioned and unlesioned sides of the brain was not what we had expected when we initiated these studies.

The pattern of glutamate release in the hemi-parkinsonian rats is also interesting, in that it has a biphasic shape, with little release in the first two minutes followed by a more rapid rise toward the plateau during the remainder of the stimulation sequence. The glutamate concentration measured by the biosensor is a function of synaptic glutamate release, glutamate reuptake by transporters, and diffusion from the site of release to the biosensor. Of these factors, the diffusion distance between the site of glutamate release and the biosensor seems most likely to cause such a delay. The delay was less between changes in glutamate levels and the onset of HFS in the STN, which we interpret as evidence that the distances between the points of glutamate release and the sensor were shorter in the more compact and less dispersed STN, though there may be local differences in the barriers to diffusion out of the STN away from the sensor that might also change the dynamics of glutamate levels differently from the GP. The increase in the glutamate concentration toward the end of the experiment could either be due to enhanced synaptic activity or, more likely, due to the saturation of the glutamate transporters involved in the uptake and removal of glutamate from the extracellular space. 

### 4.1. Issues in the Design of Feedback Control of DBS

The main focus of developing adaptive or feedback-controlled DBS has been on finding an appropriate biomarker that reflects the entire spectrum of parkinsonian symptoms [8,37]. Finding an ideal biomarker has not, thus far, been possible [9,10]. A variety of biomarkers, usually unprocessed or processed, restricted frequency domains of the LFPs measured in the STN at the site of DBS, have been used for feedback-controlled DBS, but none of them has been shown to be superior to the tried and true open loop tuning of DBS over time based on physician-observed symptom control and minimization of side effects [10]. Individual components of the spectral characteristics of LFPs in the STN turn out to be poorly correlated with the full spectrum of parkinsonian symptoms, and even the correlations between the specific symptoms (e.g., rigidity) and restricted spectral domains of LFPs are weak and variable among patients with PD [9]. The transfer functions that we identified in the 6-OHDA-treated rats tell a similar story: much as LFP activity in the STN is poorly or weakly correlated with parkinsonian symptoms, glutamate levels in the STN are poorly correlated with loss of dopaminergic neurons in the SNc. Toxigenic lesions of dopaminergic neurons did not change the transfer function between HFS in the STN and glutamate release in the STN, whereas these dopaminergic lesions significantly altered the transfer function between HFS in the STN and glutamate levels in the GP. 

We have been studying how neurotransmitter release might be used for feedback control of DBS [11]. Effective design of a feedback-control system for DBS requires that the feedback signal reflect the underlying neurophysiological abnormality so that deviation of the feedback signal from some ideal (presumably normal) level may provide an “error” signal that may be used to guide the magnitude or intensity or pattern of DBS. In the design of any feedback-controlled system, the placement of the feedback sensor is an important consideration (e.g., the thermostat for a house heating system is usually placed on an interior wall rather than a very cold external wall or the very hot wall of the furnace so as to avoid extreme temperature fluctuations within the house). The loss of dopaminergic neurons, a key finding in PD, does not seem to alter the pattern of glutamate release and uptake in the STN, and so glutamate levels in the STN are unlikely to reflect the pathology or normal functional requirements for glutamate release in the STN during DBS in the setting of reduced dopaminergic function. Glutamate levels in the GP during DBS, on the other hand, do seem to reflect the level of dopaminergic input. Therefore, it seems that glutamate levels in the GP during DBS are a better biomarker of dopaminergic neuronal loss than similar measurements in the STN. By analogy, it seems likely that indices of electrophysiological activity in the GP (action potentials, firing patterns or LFPs) will also be better correlated with dopaminergic neuronal loss and parkinsonian symptoms than LFPs in the STN. Of course, the neurotransmitter and neuroanatomical location with the highest likelihood of a good correlation with parkinsonian symptoms is likely dopamine in the striatum—even glutamate in our studies is acting as a surrogate for dopaminergic levels in the striatum [18]. In any case, the best anatomical source of feedback control for DBS in PD will likely be that nucleus that demonstrates the greatest functional neurotransmitter deficiency associated with the depletion of dopaminergic neurons in the SNc—whether the feedback-control signal is electrophysiological or chemical. 

As noted above, the sensor, whether electrical or chemical, used to control DBS should probably be placed at the neuroanatomical site where that biomarker activity is most significantly altered by the dopamine depletion in PD (so that stimulation can most effectively eradicate or normalize the abnormal electrical activity). That design criterion has not been met by the first generation of adaptive DBS devices, which detect local field potentials only at the site of stimulation (stimulating and sensing in the same location eliminates the need to pass two electrodes through the brain to implant separate recording and stimulating electrodes, and thereby reduces the surgical risk of the procedure) [38]. A similar strategy based on glutamate levels in the STN seems unlikely to work for systems designed to use glutamate levels as the feedback signal because glutamate levels are not changed appreciably at the site of stimulation in the STN by the co-existing loss of dopaminergic neurons in the SNc. That a strategy in which stimulation and feedback signal detection occur in the same anatomical location is less than ideal, is reflected in the lack of significantly improved control of the spectrum of parkinsonian symptoms achieved to date by adaptive DBS compared to the usual, tried and true, open-loop tuning of DBS [9,10].

### 4.2. Limitations of the Methods

Foremost among the limitations is that we only tried to determine what characteristics of a neurotransmitter-based feedback signal might optimally control DBS. We have not yet demonstrated that using such an optimized feedback-control system actually improves motor control in parkinsonian animals. Those experiments remain to be completed.

There are also technical limitations in our study. The glutamate biosensor is not stable over periods of extended use [11]. Although we calibrated the sensor before and after implantation, the calibration curves obtained may not be directly applicable to measurement in the brain. However, the peak concentrations of glutamate in the STN during DBS were on the order of 300 μM and slightly lower (about 200 μM) in the GP, and these values are similar to those described by others and by us in a previous study [11]. The performance of the enzyme-based biosensor tended to degrade over time and some damage to the biosensor may be sustained during explantation. The decrease in sensitivity before and after the measurement was about 30%, although the slope of the calibration curve remained unaffected. Further progress in biosensor design must be made to make the sensors more durable if neurotransmitters are to be used as chronically implanted devices. 

As noted above, the biosensors are working close to the limits of detection when measuring basal levels of glutamate in the unstimulated state. Moreover, the studies were conducted in anesthetized animals. Thus, the basal levels of glutamate measured may not be accurate. However, the dynamics of the neurotransmitter response rather than its absolute magnitude are likely to be accurate and may be more revealing of the underlying dynamical properties of the circuit. We normalized our data before attempting to construct transfer functions so that the response characteristics that we focused on were less dependent on the biosensor calibration. For this reason, we obtained regression models that were consistent from one animal to another. The glutamate dynamics derived from this analysis were not identical in the GP and the STN: the rise in glutamate levels in the GP was slower than in the STN, and the reason for this cannot be attributed to any aspect of the type of electrodes, the calibration, anesthesia, or differences in the basal levels of glutamate since these factors were similar in the GP and STN even though the glutamate dynamics differed. The ARMAX model is a global fit of the data, a global convolution of the PRBS of stimulation and the measured glutamate levels. It is difficult to look at the models and speculate about the cause of any given ripple in the fitted data other than saying it is the same or different from the pattern seen in other nuclei. The advantage of the ARMAX model is that it generates a quite accurate transfer function, but the disadvantage is that the details of the model are opaque—the parameters do not translate easily into explanations of local phenomena in the response profiles. Further studies using different modeling methods will be necessary to investigate the causes of the divergent glutamate dynamics, such as the sluggish onset of glutamate change in the GP but not the STN.

We used monophasic, constant-current stimulation in these studies, and the amount of glutamate released may vary significantly depending on whether constant-current or constant-voltage stimulation is used and on whether the stimulation is monophasic or biphasic (and therefore charge balanced) [39]. Given that we used monophasic constant-current stimulation, the glutamate levels that we measured are similar to those that others have detected using identical patterns of stimulation [11,39]. Biphasic stimulation might have resulted in lower average glutamate concentrations, and the transfer function would probably have been different had the type of current injection method differed. Nonetheless, the key results of our study remain germane to the design of any physiological feedback-controlled therapy—the anatomical site of origin of any feedback signal, whether one chooses a chemical or electrical signal, should reflect some aspect of the pathology of the disease being treated so that control of the feedback signal can be tied to correction of this “error” term and normalization of the feedback signal. 

Both the stimulating and the biosensor electrodes are relatively large (200–300 µm in diameter) and caused significant damage to the tissue. Tissue destruction may have contributed to the relatively slow dynamics of glutamate levels in both nuclei since tissue destruction probably increased the diffusion distance and the barrier to diffusion between the sites of glutamate release and the site of glutamate measurement on the surface of the biosensor. Damage could possibly be reduced by using a pair of thin tungsten or carbon fiber monopolar electrodes spaced to span a portion of the STN instead of one concentric stimulating electrode, but aligning them with the structure during surgery would add complexity to the procedure. In addition to making more durable sensors, we are trying to reduce the size of the biosensor, but this may compromise sensitivity since the surface area for glutamate detection may be reduced. Moreover, it can be difficult to load sufficient glutamate oxidase onto the surface of very small biosensors.

The variability in the level of destruction of dopaminergic neurons is also a concern. The average reduction was about 45% on the lesioned side compared to the unlesioned side, which is somewhat smaller than the reduction necessary to elicit motor changes in the 6-OHDA treated rats. We did not compare the extent of cell loss between the sham control and the 6-OHDA-treated animals on the nominally untreated side. The fact that we found differences in the DBS-glutamate transfer function in the GP suggests that even a modest reduction in the number of dopaminergic neurons is sufficient to alter the neurotransmitter responses of the brain to DBS in the STN. Splitting the lesioned animals into two groups based on the extent of neuronal death did reveal differences in the transfer function, but they were relatively small (the averaged data from one group still fit the model obtained with the other group with an r^2^ of about 0.89). The small number of the animals studied did not allow us to conduct statistical analyses to determine whether there was a specific threshold of dopaminergic neuronal loss below which glutamate dynamics in the GP were altered. In the STN, the excellent degree of fit in the animals with small (less than 30% destroyed) and large lesions (over 80% destroyed) to the same model makes it unlikely that glutamate release in the STN during DBS depends on dopaminergic innervations in any significant way.

Last, comparing glutamate release in the GP in rats to glutamate release in humans may be both confusing and misleading. In humans, the GP is subdivided into the GPe and the GPi. It is the GPi that is the target of both ablative therapy (pallidotomy) and DBS in patients with Parkinson’s Disease [5]. In rats, the GP is probably more accurately seen as the homolog of GPe. Therefore, inferences about glutamate release in the GPi in humans, based on the current work, should be made with caution.

## 5. Conclusions

The destruction of dopaminergic neurons in the SNc following the 6-OHDA treatment in rats led to altered glutamate responses in the GP, but not in the STN, during DBS in the STN. We also validated the utility of the PRBS approach in characterizing the complex dynamics of neurotransmitter levels during DBS and showed that changes in the transfer function obtained from such an analysis may be used to study pathological changes in a neuronal circuit. The finding that the glutamate response to DBS in the STN is altered in the GP in the 6-OHDA-treated animals makes the glutamate concentration at this site a possible feedback-control signal for a closed-loop DBS device in PD. A feedback-controlled or adaptive stimulator removes the need for subjective adjustment of the stimulator parameters, reduces the possibility of eliciting unwanted side effects from over- or under-stimulation, and could adapt dynamically to changes in the brain that may occur in response to changes in motor activity, waking and sleeping state, or local tissue around the stimulating electrode during chronic use of DBS, or progression of PD.

## Data Availability

The data presented in this study are available on request from the corresponding author.
